# Enhancing biomedical data validity with standardized segmentation finite element analysis

**DOI:** 10.1038/s41598-022-13961-0

**Published:** 2022-06-14

**Authors:** Matthew A. Wysocki, Scott Doyle

**Affiliations:** Department of Pathology and Anatomical Sciences, University at Buffalo, Buffalo, 14203 USA

**Keywords:** Biomedical engineering, Anatomy, Medical research, Physics

## Abstract

Finite element analysis is a powerful computational technique for augmenting biomedical research, prosthetics design, and preoperative surgical assessment. However, the validity of biomechanical data obtained from finite element analysis is dependent on the quality of the preceding data processing. Until now, little information was available about the effect of the segmentation process on finite element models and biomechanical data. The current investigation applied 4 segmentation approaches to 129 femur specimens, yielding a total of 516 finite element models. Biomechanical data including average displacement, pressure, stress, and strain were collected from experimental groups based on the different segmentation approaches. The results indicate that only a 5.0% variation in the segmentation process leads to statistically significant differences in all 4 biomechanical measurements. These results suggest that it is crucial for consistent segmentation procedures to be applied to all specimens within a study. This methodological advancement will help to ensure that finite element data will be more accurate and that research conclusions will have greater validity.

## Introduction

### Role of segmentation in 3D model analysis

Accurate use of 3D modeling techniques in medical education, biomedical research, and clinical applications requires having comprehensive understanding of how data-processing steps alter 3D anatomical models. One of the most critical data-processing steps in 3D model generation is segmentation; the process of manually or automatically extracting voxels from regions of interest^1^. In particular, 3D model structure can be dramatically altered by: numerous aspects of manual segmentation that introduce inconsistencies between specimens, automatic segmentation that is based on different algorithms, artifact inclusion during manual and automatic segmentation, failure to extract portions of the structure of interest, and variations in the parameters that are applied to automatic segmentation algorithms^2–4^. The influence of these image segmentation inconsistencies on 3D model structure are consequential for medical education (e.g., gross anatomy instruction), research, and clinical applications.

### Segmentation discrepancies lead to inconsistent analysis

**Figure 1:**
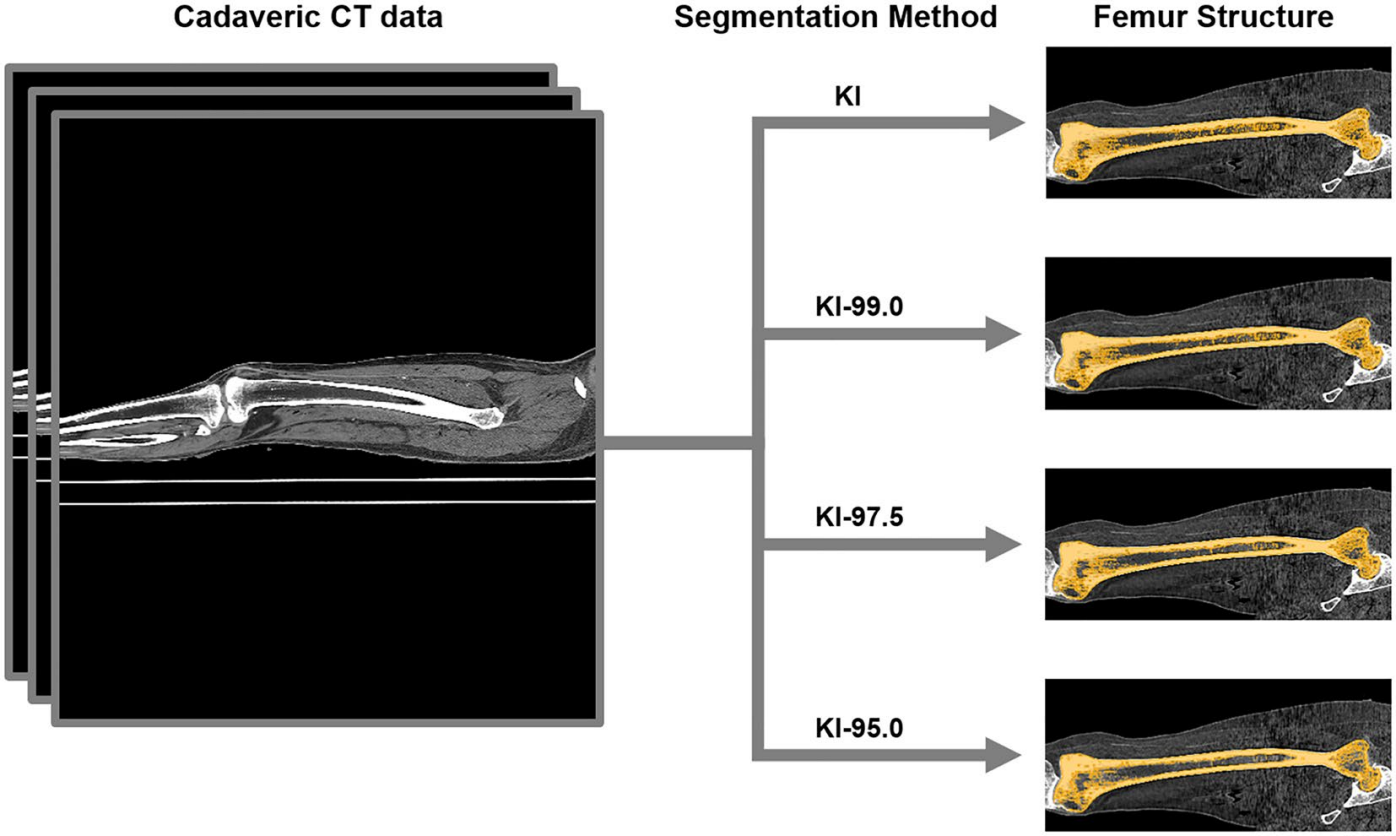
Overview of cadaveric CT data segmentation used to extract series of 3D anatomical models of femur structure. Segmentation methods: Kittler–Illingworth algorithm (KI), 99.0% intermediate intensity value thresholding approach (KI-99.0), 97.5% intermediate intensity value thresholding approach (KI-97.5), 95.0% intermediate intensity value thresholding approach (KI-95.0). Segmented femur osteological structure is shown in orange, coronal plane.

Given that segmentation procedures ultimately determine the 3D model data that serve as the foundation for achieving scientific discoveries in the fields of morphology, biomechanics, and medicine, it is important to examine how different segmentation procedures relate to variation in 3D models and associated quantitative data^5–8^. Huotilainen et al.^1^ demonstrated that variations in the DICOM-to-STL conversion step of processing computed tomography (CT) data from the same individual significantly change the morphology of subsequent 3D anatomical models. Variations in CT data segmentation methods have even been found to generate 3D anatomical models of osteological structure that are superficially similar, but quantitatively distinct in multiple morphological measurements^9^. Although it is evident that inconsistencies in the CT data segmentation process influence quantitative morphological data, it is not known how variation in the segmentation (Fig. 1) process changes biomechanical data obtained from finite element (FE) analysis.

### Previous work in understanding 3D modeling inconsistencies

The FE method is a highly versatile and powerful technique for carrying out in silico biomechanical testing of complex biological structures^10^. However, advancing FE analysis of biological structures is especially challenging because the resulting biomechanical data are dependent on the accuracy of geometric reconstruction (i.e., specimen morphology), material properties, and boundary conditions (i.e., experimental loading and 3D model constraints)^6^. Inaccuracies in any of these parameters are potentially compounded in the FE output data^11^. Indeed, previous studies have revealed inconsistencies between the biomechanical data obtained from FE analysis and the data collected from in vivo experiments; disparities that afflict both the magnitude and the orientation of biomechanical data^6,11,12^.

### Significance of standardized 3D model generation

Fully-understanding the effect of segmentation and other data processing procedures on FE data is crucial for medical research and clinical applications. 3D anatomical models serve as the structural basis for the 3D printed prosthetics used in reconstructive surgery^13^. Similarly, 3D models of anatomical structures are used in FE analysis to determine the biomechanical characteristics of those prosthetics in order to assess their functional properties, verify their safety for patients, and develop treatment approaches^14–16^. In addition, 3D data are commonly used to create patient-specific physical models that surgeons utilize for preoperative assessments of alternative surgical strategies that reduce the time requirements and surgical risks^1,17^. The current investigation evaluates how different segmentation approaches influence FE analysis of anatomical structures in order to enhance this method for improving research findings and clinical outcomes.

## Methods

### Data

The experiments of this study were performed in accordance with all relevant guidelines and regulations, and with the approval of the Jacobs School of Medicine and Biomedical Sciences, University at Buffalo, the State University of New York. The current investigation uses digital imaging data from cadaveric donors. No data were collected from living human subjects. The use of these cadaveric CT data in this investigation and the experimental protocol of the study were approved by the UB Anatomical Gift Program and the UB Department of Pathology and Anatomical Sciences.

All cadaveric donors provided informed consents prior to death directing that after death, their bodies were to be given to University at Buffalo, the State University of New York. These declarations of consent specifically state that their bodies are to be donated to UB as unrestricted gifts for the purposes of medical study and research. All of the digital cadaveric data used in the study were anonymized. Furthermore, all personal identifiers have been removed from every section of this manuscript and from the supplementary information files.

Thorough examination of how FE data are influenced by the segmentation process was carried out using CT data provided by the Anatomical Gift Program of the University at Buffalo (UB). The cadaveric CT-data from all anatomical donors was collected using the facilities of the UB Clinical and Translational Science Institute (CTSI) Center for Biomedical Imaging. Series of 3D anatomical models were extracted from each of 129 femur specimens with one dataset consisting of left femurs (n = 66) and a second dataset comprised of right femurs (n = 63). Overall, there was a total of 516 anatomical models for the ensuing biomechanical assessments. Anatomical donors with joint replacements, surgical pins, and advanced osteoarthritis were excluded from the sample.

### CT data segmentation

The segmentation of cadaveric CT data from both datasets was carried using 4 different segmentation approaches. The segmentation approaches consisted of: the Kittler–Illingworth (KI) method, the intermediate 99.0% of the Kittler–Illingworth intensity values approach (KI-99.0), the intermediate 97.5% of the Kittler–Illingworth intensity values approach (KI-97.5), and the intermediate 95.0% of the Kittler–Illingworth intensity values approach (KI-95.0)^18^. The assessments of the KI and KI-99.0, KI and KI-97.5, and KI and KI-95.0 segmentation experimental groups represent variations in the segmentation intensity values of 1.0%, 2.5%, and 5.0%, respectively.

Although superficially comparable to one another, these 4 strategies differed slightly in their capacities to extract the osteological structure of interest. The KI method was the best-suited strategy for maximizing osteological structure extraction, whereas the KI-95.0 approach was the technique that excluded the greatest amount of preserved soft tissue and imaging artifacts. All segmentation was completed in 3D Slicer; an open-source imaging platform^2^. The segmented femur structures were exported as stereolithography (.stl) files for 3D mesh processing to permit biomechanical analysis.

### Data standardization

The 4 different segmentation approaches yielded 3D anatomical models that were visually indistinguishable. In preparation for FE analysis, the femur 3D models were processed with several data standardization steps. In the open-source mesh processing software MeshLab, the 3D models were standardized with automated functions: isolated piece removal, non-manifold edge repair, duplicate face removal, intersecting face removal, T-vertices removal, surface mesh hole closure, and processing with a Screened Poisson algorithm^19,20^. Furthermore, the femur 3D anatomical models were standardized as cropped femoral head 3D anatomical models to permit biomechanical analysis without introduction of a potential second variable (i.e., 3D anatomical model simplification), and to enable greater consistency in force and constraint assignment in FE analysis. Additionally, the femoral head 3D anatomical models were uniformly oriented along the Z-axis in freely available MeshMixer^21^.

### Finite element (FE) analysis

FE analysis of 3D anatomical models was carried out in FEBio, which is an open-source software environment tailored for biological applications^22^. The triangular surface mesh anatomical models were converted to solid mesh anatomical models for biomechanical evaluation. These solid meshes were made up of nodally integrated tetrahedral elements that ensure greater performance than typical constant strain tetrahedral elements^23^. Akin to previous biomechanical testing, the femoral head 3D anatomical models had a Young’s modulus of 16800 MPa and Poisson’s ratio was 0.3^14,15,24–26^. Although bone as a material can display additional characteristics, for the purpose of FE analysis in the present study, the 3D anatomical models were assigned the isotropic elastic material type that has been utilized in previous FE studies of the femur and other osteological structures^14,15,26–29^. All 3D anatomical models were subjected to 1800N compressive loading assigned to the proximal aspect of the femoral head to simulate the standing joint reaction force of one leg; consistent with prior research^15,31^. The boundary conditions were set to restrict x, y, and z displacement at the inferior surface (i.e., junction of the femoral head and femoral neck).

Four measurements of biomechanical performance commonly used in biological FE studies were obtained from each 3D anatomical model (S1 Text). These consisted of average displacement (i.e., total nodal displacement), average pressure, average stress (i.e., von-Mises stress), and average Lagrange strain values of the tetrahedral elements^30,32^. Average quantities from across each 3D model were used for each of these types of biomechanical data in order to acquire an overview of the relationship between the CT data segmentation process and FE model data. Moreover, average values for the measurements, rather than point or cross-section values, were utilized to avoid the possibility of local minima or maxima in biomechanical data from potentially overestimating differences between 3D models generated from different segmentation approaches. Ultimately, statistical analysis of these biomechanical performance data were carried out to determine if the FE data were significantly influenced by the segmentation process.

### Ethical approval

This study was performed in accordance with the ethical standards as laid down in the 1964 Declaration of Helsinki and its later amendments or comparable ethical standards. The donor cadavers and the associated digital data are the property of the State University of New York at Buffalo. All informed consents were obtained from the donors prior to death by the University at Buffalo Anatomical Gift Program. Great care has been taken in this study to ensure that all potentially identifiable digital cadaveric data have been removed to make all data anonymous.

## Results

### Dataset 1: left femur sample

**Figure 2:**
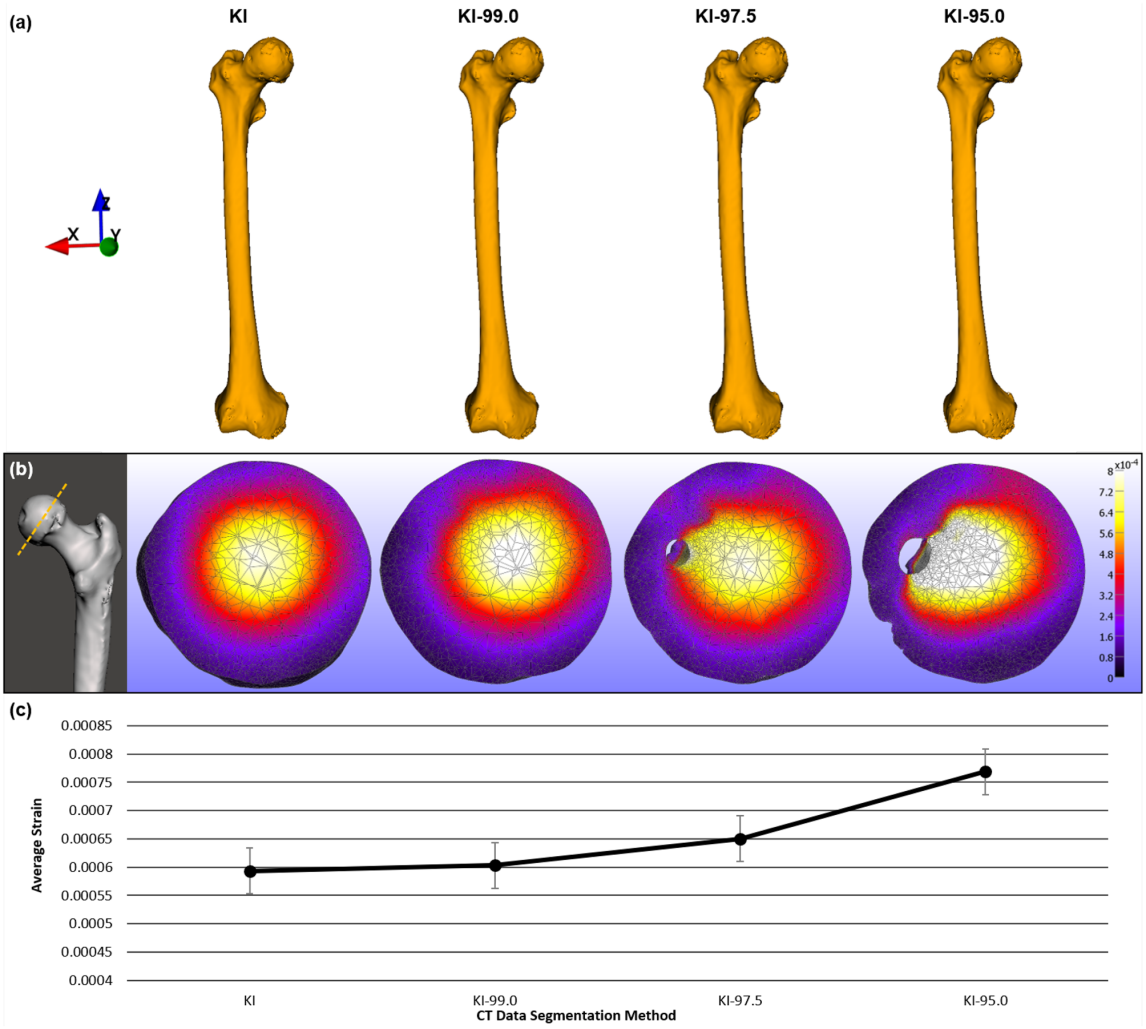
Visually comparable 3D anatomical models yield different biomechanical data. (**a**) 3D models of the same right femur specimen generated using 4 different segmentation methods (anterior view). (**b**) Cross-sections of femoral head revealing hidden internal morphological variations and heatmaps showing strain data variation. (**c**) Average strain data (n = 63) from finite element models generated with 4 segmentation methods. 3D mesh visualizations generated with MeshMixer 3.5.474, www.meshmixer.com; data heatmaps generated with FEBio Studio 1.0.0, https://febio.org.

The average displacement results of the left femur dataset (S2 Text; S1 Table) showed variation across the segmentation experimental groups, with average displacement values increasing as segmentation intensity value parameters decreased. The t test results indicate that these differences in average displacement were statistically significant between the KI and KI-97.5 segmentation experimental groups (p = 0.0095) and the KI and KI-95.0 segmentation experimental groups (p = 0.0413), whereas the results were not statistically significant for the KI and KI-99.0 results (p = 0.5356) (see Fig. 2). Statistically significant differences in the average pressure data (S2 Table) were evident between the KI and KI-95.0 segmentation experimental groups (p = 0.0006), but the pressure data differences were not statistically significant for the KI versus KI-99.0 (p = 0.4859) or the KI versus KI-97.5 segmentation experimental groups (p = 0.0799).

Similarly, the results for average stress (S3 Table) showed that the KI and KI-95.0 experimental group data were significantly different from each other (p = 0.0020), however the data disparities of the KI and KI-97.5 (p = 0.0577), as well as KI and KI-99.0 (p = 0.4968), experimental groups were not significantly different. Dissimilarities were also evident in the average strain data (S4 Table) of the four segmentation experimental groups. Specifically, the results showed that the difference between KI and KI-95.0 strain data was statistically significant (p = 0.0021), whereas the strain data variations were not significantly different for KI and KI-97.5 (p = 0.0577) or the KI and KI-99.0 (p = 0.4961) experimental groups. Overall, the KI and KI-95.0 segmentation approaches generated 3D anatomical models that had significantly different FE analysis data for all 4 types of biomechanical performance measurements (see Table 1).

**Table 1:** Left femur dataset summary of t test results from analyses of biomechanical data between the different segmentation experimental groups.

	KI vs KI-99.0	KI vs KI-97.5	KI vs KI-95.0
Displacement	0.5356	**0.0413**	**0.0095**
Pressure	0.4859	0.0799	**0.0006**
Stress	0.4968	0.0577	**0.0020**
Strain	0.4961	0.0577	**0.0021**

p values shown. Statistical significance indicated in bold.

### Dataset 2: right femur sample

**Figure 3:**
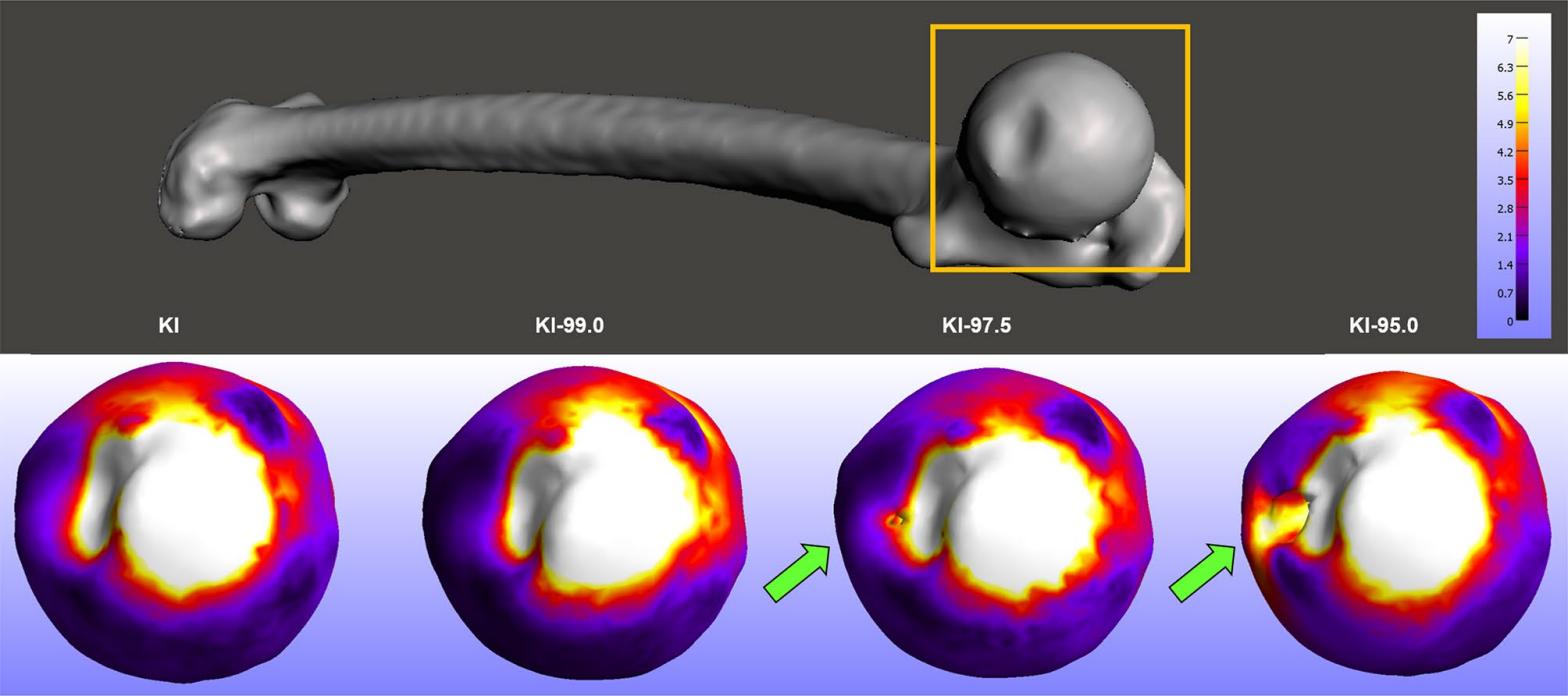
Dissimilarities in regional stress data of finite element models of a right femur specimen generated using 4 different segmentation methods. Arrows indicate a localized development of high stress (MPa) values. 3D mesh visualizations generated with MeshMixer 3.5.474, www.meshmixer.com; data heatmaps generated with FEBio Studio 1.0.0, https://febio.org.

In general, the biomechanical data from the right femur dataset were quite similar to those from the left femur dataset. The data exhibited variation across segmentation experimental groups of all four biomechanical measurements (Fig. 3). Greater average values occurred for segmentation experimental groups with narrower intensity value parameters. In particular, the differences in average displacement data (S5 Table) were statistically significant (p = 0.0023) between KI and 95.0-KI. The t test results also showed that displacement data differences were not statistically significant for KI versus KI-97.5 (p = 0.0583) or for KI versus KI-99.0 (p = 0.5158). Statistical analysis of the average pressure data (S6 Table) indicated that the measurement differences were significant between the KI and KI-95 segmentation experimental groups (p = 0.0002), whereas the average pressure data variation was not statistically significant between the KI and KI-97.5 experimental groups (p = 0.0568) or between the KI and KI-99.0 experimental groups (p = 0.6017).

Disparities in the average stress data (S7 Table) between the KI and KI-95.0 segmentation experimental groups (p =<0.000), as well as those between the KI and KI-97.5 experimental groups (p = 0.0423), were statistically significant. Alternatively, the results (p = 0.6589) for KI and KI-99.0 indicated that the differences in average stress data were not statistically significant. The results showed that the differences in average strain data (S8 Table) of the KI and KI-95.0 segmentation experimental groups (p = 0.0019) and of the KI and KI-97.5 experimental groups (p = 0.0427) were statistically significant, but the differences between the KI and KI-99.0 average strain data were not statistically significant (p = 0.6604). Table 2 displays a summary of how the right femur biomechanical data of all 4 measurement types were significantly different between the KI and KI-95.0 segmentation experimental groups.

**Table 2:** Right femur dataset summary of t test results from analyses of biomechanical data between the different segmentation experimental groups.

	KI vs KI-99.0	KI vs KI-97.5	KI vs KI-95.0
Displacement	0.5158	0.0583	**0.0023**
Pressure	0.6017	0.0568	**0.0002**
Stress	0.6589	**0.0423**	**< 0.000**
Strain	0.6604	**0.0427**	**0.0019**

p values shown. Statistical significance indicated in bold.

### Overall finite element (FE) analysis results

The data from FE analysis of 3D anatomical models of the left femur dataset (n = 66) and right femur dataset (n = 63) were comparable across all of the biomechanical measurements, as shown in Table 3. The values of these biomechanical performance data increase in association with reductions in the CT-data segmentation intensity value parameters utilized to generate the 3D anatomical models. The results show that the biomechanical performance data are marginally dissimilar between the KI and KI-99.0 segmentation experimental groups, whereas the biomechanical data differences become more pronounced between the KI and KI-97.5 segmentation experimental groups, and between the KI and KI-95.0 segmentation experimental groups.

**Table 3:** Differences in biomechanical data from finite element (FE) models of the same specimens, but generated with 4 different segmentation methods.

	KI		KI-99.0		KI-97.5		KI-95.0	
	L	R	L	R	L	R	L	R
Displacement	0.0067	0.0063	0.0071	0.0066	0.0109	0.0113	0.0192	0.0187
Pressure	2.0802	2.0162	2.1283	2.0526	2.2138	2.1633	2.4172	2.3710
Stress	7.7947	7.6379	7.9737	7.7652	8.4279	8.3705	9.6441	9.4757
Strain	0.0006	0.0006	0.0006	0.0006	0.0007	0.0007	0.0007	0.0008

Mean displacement (mm), pressure (MPa), stress (MPa), and strain values of left (L) and right (R) femur datasets shown.

**Figure 4:**
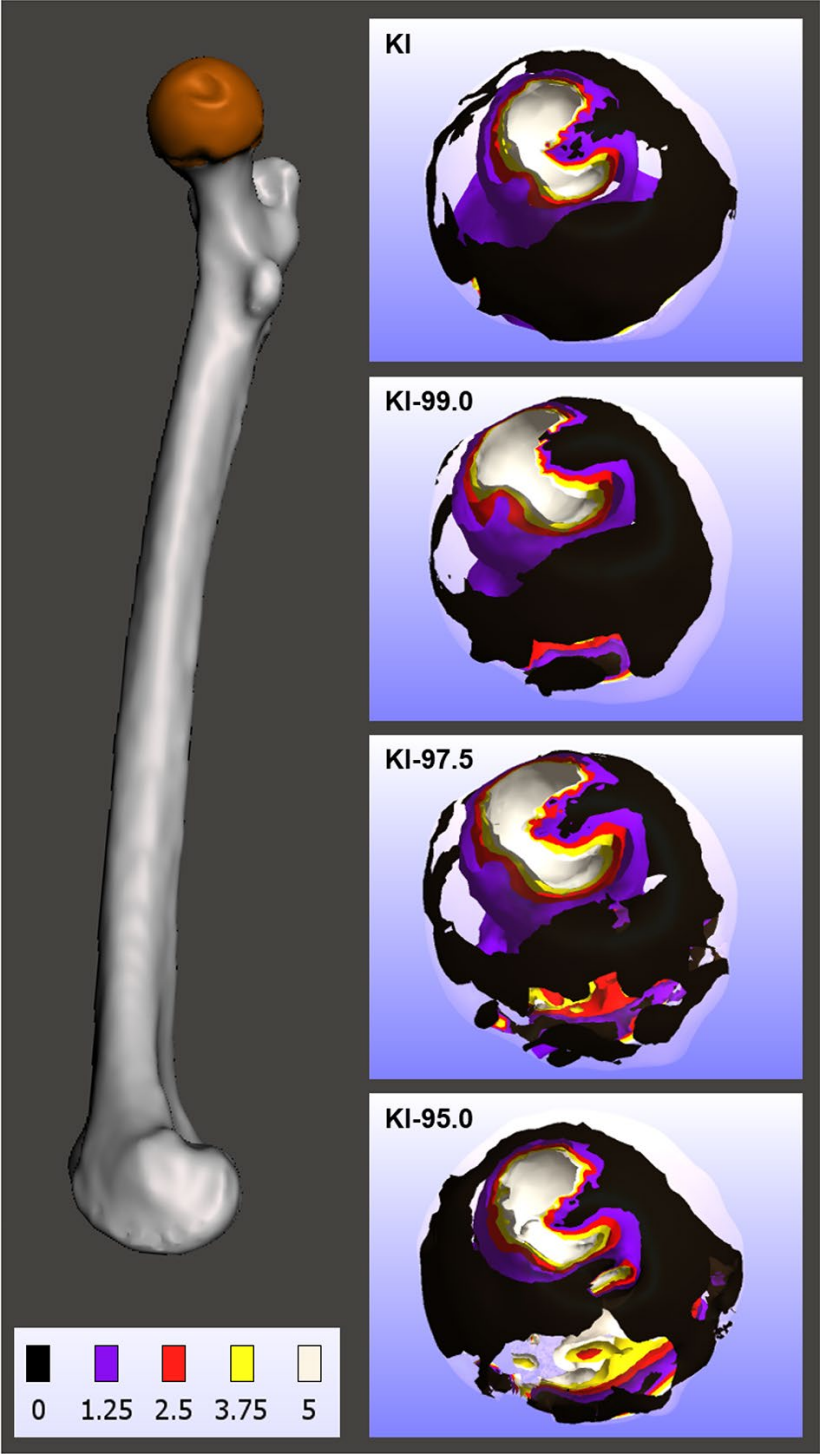
Finite element data isosurfaces showing variation in pressure (MPa) value distributions of right femur 3D anatomical models generated using 4 different segmentation methods (medial view). 3D mesh visualizations generated with MeshMixer 3.5.474, www.meshmixer.com; data heatmaps generated with FEBio Studio 1.0.0, https://febio.org.

The disparities in values of each biomechanical measurement (i.e., displacement, pressure, stress, and strain) between the KI and KI-95.0 segmentation experimental groups were statistically significant for all 4 measurements; a finding that was evident in both the left femur dataset and the right femur dataset. When it comes to the differences between biomechanical data of the KI and KI-97.5 segmentation experimental groups, this magnitude of variation in the segmentation process approached the limit to which segmentation can be modified without substantially degrading FE data. The results from the left femur dataset show statistically significant differences in the displacement data of the KI and KI-97.5 segmentation experimental groups. Moreover, the right femur dataset results indicate that stress and strain biomechanical measurements had statistically significant differences between the the KI and KI-97.5 segmentation experimental groups (Fig. 4).

## Discussion

Prior to the current investigation, little information was available concerning the effect of the segmentation process on FE data. Previous studies had documented how very subtle variations in segmentation procedures could lead to significantly different 3D structure and quantitative morphological data, but whether or not quantitative biomechanical data (i.e., FE data) are significantly altered by slight variations in the segmentation process was not known^1,9^. The results of the present study indicate that even though variations in segmentation procedures can appear negligible during visualization of CT data and yield visually comparable 3D anatomical models, these disparities alter 3D anatomical model structure enough to significantly change biomechanical performance.

In addition to the overall results in which average biomechanical measurements differ between the segmentation experimental groups, regional variations in biomechanical data were apparent across each specimen for 3D anatomical models generated using different segmentation approaches. For instance, considerable variation in regional strain data were evident between experimental groups; the KI-95.0 3D models exhibited relatively high strain levels emerging inferior to fovea capitis, which did not occur in the KI or the KI-99.0 3D models. Similarly, regional variations in pressure data distribution are evident across 3D anatomical models generated with different segmentation methods. Thus, subtle variations in the segmentation process that alter the degree of osteological tissue included within 3D anatomical models can introduce regional structural variations that alter biomechanical performance.

The current study utilizes segmentation approaches that are forms of the KI segmentation method because this segmentation method is: (1) mathematically-based, (2) publicly available in 3D Slicer, and (3) previously demonstrated to effectively delineate preserved soft tissue from bone tissue while still extracting intact femur osteological structure^2,9,33,34^. The purpose of this investigation, however, is not to emphasize these segmentation methods over others, but to formally test the influence of variation (inconsistency) in the segmentation process on biomechanical data. Studies using FE analysis rarely document and report the methodological details of segmentation that were used to generate the underlying 3D models. As well, the 3D models within a given research study may be generated from manual segmentation methods as opposed to standardized, automated segmentation methods; potentially introducing substantial variation into the FE data. What is more, FE research studies commonly report conclusions that are based on data from very small sample sizes because of the time-intensive nature of the FE method. Given that the current study examines subtle ($\le 5\%$) variation in a standardized, mathematically-based segmentation method applied to datasets with robust sample sizes, the results suggest that greater disparities in FE data occur when less stringent segmentation methods are used.

Considering that the femur model system represents a less complex osteological structure, these findings are likely applicable to FE data obtained from numerous other structures. It is expected that FE analysis of bones with fine structures and greater geometric complexity (abundant processes, spines, tubercles, epicondyles, ridges, crests, facets, alveoli, foveae, grooves, canals, canaliculi, and foramina) would be even more dramatically altered by variations in the segmentation process. This is consistent with previous results, which have shown that strain values of thin bone FE models are influenced by voxel size variation^35^. The current results suggest that inconsistencies in the segmentation process may be especially relevant to FE studies that involve intricate structures because these are more apt to be vastly altered by subtle changes to voxel inclusion or exclusion in the 3D models. Furthermore, these findings may also be applicable to other types of FE models used in biomedical engineering (e.g., simulations of organ deformation, muscle mechanics, blood flow, thrombus formation, and medical implant biodegradation)^36–42^.

The FE method offers many advantages including the collection of biomechanical data from medical imaging data, non-destructive biomechanical data collection from anatomical specimens, and flexible experimental designs, but the concerns over FE data validity and reliability show that the technique needs to be refined in order to avoid erroneous results and conclusions^6,10–12^. Both FE datasets indicate that merely a 5.0 percent change or less in CT data segmentation intensity values leads to 3D anatomical models of osteological structure that will have statistically significant differences in 4 biomechanical performance measurements. These results indicate that minor disparities in segmentation approaches may introduce inconsistencies that significantly change FE data and potentially lead to erroneous research conclusions. Therefore, it is strongly recommended that uniform segmentation processing of CT data is applied to all specimens within a sample and that these protocols are fully-documented within literature to advance the FE method and its validity as a powerful computational technique in biomedical research.

## Supplementary Information

Supplementary Information 1.

Supplementary Information 2.

Supplementary Information 3.

Supplementary Information 4.

Supplementary Information 5.

Supplementary Information 6.

Supplementary Information 7.

Supplementary Information 8.

Supplementary Information 9.

Supplementary Information 10.

## Data Availability

All of the data used to derive the results presented within this manuscript are made freely available to the public in accordance with Scientific Report’s data availability requirements.
