# Supplementary material for: Enhancing biomedical data validity with standardized segmentation finite element analysis
Source: Sci Rep. 2022 Jun 14;12:9860. doi: 10.1038/s41598-022-13961-0 (PMC9198234; doi:10.1038/s41598-022-13961-0)
Supplement: Supplementary file 2 — Supplementary Information 2. [file 41598_2022_13961_MOESM2_ESM.pdf]

S1 Text.

The hyperelastic strain-energy function [1, 2]:

$$W = \frac{1}{2}\lambda(tr\mathbf{E})^2 + \mu(\mathbf{E} : \mathbf{E}) \tag{1}$$

where the Lamé parameters are λ and μ:

$$\lambda = \frac{vE}{(1+v)(1-2v)}, \quad \mu = \frac{E}{2(1+v)}$$

Average displacement (i.e., total nodal displacement) across each 3D anatomical model was measured [5]:

$$||d|| = \sqrt{(x_2 - x_1)^2 + (y_2 - y_1)^2 + (z_2 - z_1)^2} \tag{2}$$

The average pressure of each 3D anatomical model was measured with the formula [2]:

$$p = \frac{1}{3}tr\sigma \tag{3}$$

Average stress (i.e., von-Mises stress) of each 3D anatomical model was calculated with the formula [2]:

$$\sigma_Y(U) = \sqrt{\frac{3}{2}\sigma_{dev} : \sigma_{dev}} \tag{4}$$

Average strain was calculated via the Green-Lagrangian strain tensor [2, 5]:

$$\mathbf{E} = \frac{1}{2}(\mathbf{C} - \mathbf{I}) \tag{5}$$

References

[1] Steve A Maas, Benjamin J Ellis, Gerard A Ateshian, and Jeffrey A Weiss. Febio: finite elements for biomechanics. *Journal of biomechanical engineering*, 134(1), 2012.

[2] Steve A Maas and Jeffrey A Weiss. Febio theory manual. *http://mrl.sci.utah.edu/software/febio.*, 2021.

[3] Lin Tianye, Yang Peng, Xu Jingli, Wei QiuShi, Zhou GuangQuan, He Wei, and Zhang Qingwen. Finite element analysis of different internal fixation methods for the treatment of pauwels type iii femoral neck fracture. *Biomedicine & Pharmacotherapy*, 112:108658, 2019.

[4] Jan Van Houcke, Ashwin Schouten, Gunther Steenackers, Dirk Vandermeulen, Christophe Pattyn, and Emmanuel A Audenaert. Computer-based estimation of the hip joint reaction force and hip flexion angle in three different sitting configurations. *Applied ergonomics*, 63:99–105, 2017.

[5] Steve A Maas, Jeffrey A Weiss, and Gerard Ateshian. Febio user's manual. *http://mrl.sci.utah.edu/software/febio.*, 2020.
