# Supplementary material for: Enhancing biomedical data validity with standardized segmentation finite element analysis
Source: Sci Rep. 2022 Jun 14;12:9860. doi: 10.1038/s41598-022-13961-0 (PMC9198234; doi:10.1038/s41598-022-13961-0)
Supplement: Supplementary file 4 — Supplementary Information 4. [file 41598_2022_13961_MOESM4_ESM.docx]

Supplementary information

S1-S8 tables provide the detailed information from the Finite Element (FE) analysis data collected from 2 femur datasets. Overall, 4 biomechanical measurements were collected from each dataset during compressive loading experiments simulating standing joint reaction force. Left femur dataset (n=66). Right femur dataset (n=63).

Anatomical donors with joint replacements, surgical pins, and other artificial medical structures were excluded from the sample. Similarly, individuals with advanced osteoporosis and osteoarthritis were excluded from the sample.

Cadaveric donor data note: All data in this study are from donors who provided informed consent prior to death and great care has been taken to ensure that potentially identifiable information has been removed from the digital data to ensure donor anonymity. (See main paper text for full description).

Legend: CT-data segmentation method descriptions. KI = robust Kittler-Illingworth method. KI-99.0 = Kittler-Illingworth intermediate 99.0 method. KI-97.5 = Kittler-Illingworth intermediate 97.5 method. KI-95.0 = Kittler-Illingworth intermediate 95.0 method.

S1 Table. Displacement data, left femur dataset.

S2 Table. Pressure data, left femur dataset.

S3 Table. Stress data, left femur dataset.

S4 Table. Strain data, left femur dataset.

S5 Table. Displacement data, right femur dataset.

S6 Table. Pressure data, right femur dataset.

S7 Table. Stress data, right femur dataset.

S8 Table. Strain data, right femur dataset.
